# Up-regulation of fibroblast growth factor 19 and its receptor associates with progression from fatty liver to hepatocellular carcinoma

**DOI:** 10.18632/oncotarget.10750

**Published:** 2016-07-21

**Authors:** Yan Li, Weizhong Zhang, Anne Doughtie, Guozhen Cui, Xuanyi Li, Harshul Pandit, Yingbin Yang, Suping Li, Robert Martin

**Affiliations:** ^1^ Division of Surgical Oncology, Department of Surgery, School of Medicine, University of Louisville, Louisville, KY, 40202, USA; ^2^ Department of Hand Surgery, China-Japan Union Hospital, Jilin University, Changchun, Jilin, 130022, China; ^3^ Department of Hepatology, Cancer Center, The First Hospital of Jilin University, Changchun, 130021, China

**Keywords:** hepatocellular carcinoma, FGF19, FGFR4, cancer stem cell

## Abstract

**Background:**

Human fibroblast growth factor 19 (FGF19), its receptor (FGFR4) and EpCAM play an important role in cell proliferation, differentiation, motility, and overexpression have been linked to hepatocellular carcinoma (HCC). The aim of this study was to evaluate the FGF19 signals responsible for the progression of HCC arising from fatty liver.

**Results:**

FGF19 level was significantly increased in the HCC patients' serum compared to non-HCC controls. The IHC results demonstrated significant increases of protein expressions of FGF19, FGFR4 and EpCAM in specimens with fatty liver, NASH, cirrhosis, and HCC compared to healthy liver tissue. There was a significant positive correlation between the protein expressions (FGF19, FGFR4, and EpCAM) and histopathologic changes from FL to HCC. Furthermore, FGF19 was positively correlated with FGFR4 and with EpCAM.

**Materials and Methods:**

FGF19 protein levels in serum and tissues were determined by ELISA assay. The FGFR4, and EpCAM expression and tissue distribution were further evaluated by immunohistochemical staining in tissue array samples. FGF19, FGFR4 and EpCAM expressions between the different histologic stages of fatty liver steatohepatitis-cirrhosis-HCC carcinogenesis sequence were compared to healthy hepatic tissue.

**Conclusions:**

Overexpression of FGF19/FGFR4 significantly correlated with EpCAM as a marker of hepatic cancer stem cells within the fatty liver-steatosis-cirrhosis-HCC sequence.

**Impact:**

This is the first study to elucidate FGF19/FGFR4 signaling in favor of HCC cells developing as indicated by increased EpCAM within the carcinogenesis sequence from fatty liver to hepatocellular carcinoma. Our study has the potential to yield novel and cost effective screening strategies for HCC patients.

## INTRODUCTION

Hepatocellular carcinoma (HCC) is a highly aggressive solid tumor that develops in the context of chronic liver disease and cirrhosis [1]. HCC is histologically the cause of 70–90% of primary liver cancers, resulting in the second leading cause of cancer deaths among men and the sixth leading cause of cancer deaths amongst women worldwide [2]. It is well known that Hepatitis B, Hepatitis C, diabetes, non-alcoholic fatty liver disease, aflatoxin, alcohol, cigarette smoke, and androgenic steroids are significant risk factors for HCC [3–5]. In westernized countries, the obesity epidemic has led to multiple studies demonstrating the link between fatty liver disease, chronic inflammation or steatohepatitis, and hepatocarcinogenesis [6]. Additionally, obesity is associated with both increased risk and worse outcomes for many cancers including HCC [7], and it is likely that with the increasing rate of obesity that a corresponding increase in HCC arising from non-alcoholic fatty liver disease may be seen in the future [8].

It is estimated that up to one third of the population have evidence of fatty liver disease related to obesity [9] and metabolic syndrome [10]. Approximately one third of HCC patients do not have an identifiable viral etiology such as a hepatitis B or hepatitis C infection as a cause for their cirrhosis, and clinicians are more frequently diagnosing HCC resulting from idiopathic or non-alcoholic cirrhosis [11]. A growing body of knowledge describes this process stemming from the initial state of fatty liver disease progressing on to nonalcoholic steatohepatitis (NASH) and eventually to cirrhosis with a subpopulation transforming to HCC [12]. Because an increasing number of patients are being diagnosed with NASH, a known risk factor for hepatocellular carcinoma, a more comprehensive understanding of this disease mechanism is critical to developing new clinical strategies for early detection, treatment, and even prevention.

The fibroblast growth factor subfamily functions as endocrine factors or hormones regulating various cellular processes involving glucose and lipid metabolism, as well as bile acid synthesis and insulin-like effects in the liver [13, 14]. Several studies have shown that both fibroblast growth factor 19 (FGF19) mRNA and protein are widely distributed in human tissues where they play an important role in cell proliferation, differentiation, and motility [15]. Recently, it has been demonstrated that overexpression of human FGF19 and its murine ortholog (FGF15) lead to dysplasia and HCC [16, 17]. In the HCC disease state, high levels of FGF19 and FGFR4 have been associated with poor outcome in HCC patients [18, 19]. Mechanistically, it has been reported that effects of FGF19 is mediated through its associated fibroblast growth factor 4 (FGFR4), being implicated the activation of a number of downstream signaling pathways including Wnt/β-catenin pathways [20, 21]. Overexpression of epithelial cell adhesion molecule (EpCAM), an important component of Wnt/β-catenin signal, has been implicated as a prognostic biomarker and is associated with poor prognosis [22]. EpCAM positive HCC is more resistant to single agent chemo- or immunotherapy and therefore more likely to recur [23]. It has been speculated that HCCs displaying increased levels of EpCAM possess a more aggressive biologic behavior because they are a direct population derivative of hepatic cancer progenitor cells [24].

We hypothesize that a specific microenvironment may foster the proliferation of hepatic stem cells as seen by the significant increase in FGF19, FGFR4, and EpCAM protein levels in HCC specimens. The aim of this study is to investigate the molecular components of the signaling pathways that are responsible for the development and progression of HCC and to provide the evidence for the clinical activity of FGF19, FGFR4, and EpCAM in patients with HCC arising from fatty liver disease.

## RESULTS

### Subject selection and histological confirmation

**Figure 1:**
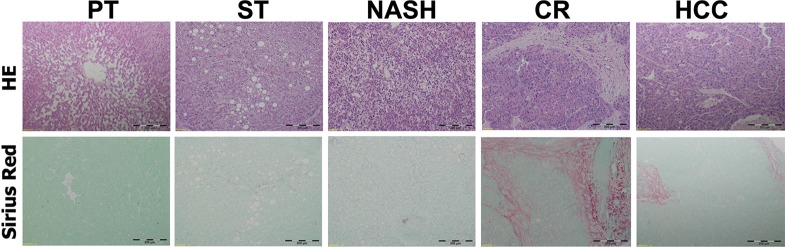
Histologic appearances of peritumoral normal and ST-NAHS-cirrhosis-HCC progression Upper: Representative histologic changes from different stages by Hematoxylin and Eosin (H&E) staining, magnification: 40×. Lower: Representative histologic changes to reveal fibrosis and cirrhosis by Sirius Red staining, magnification: 40×. PT: peritumoral tissues; ST: steatosis with diffuse lipid deposition; NASH: lipid deposition with inflammatory cells infiltration; CR: cirrhosis with regenerative nodule; HCC: hepatocellular carcinoma.

In the study, 24 subjects, 13 male (54.2%) and 11 female (45.8%) with a median age of 67, ranging from 41 to 84 years old who all had a clinical diagnosis of hepatocellular carcinoma and underwent liver resection. The median number of tumors per subject was 1 ranging up to 4, with some of the tumors being a large confluence of multiple smaller tumors. The median size of tumors was 7.75 centimeters (cm), while most of the tumors were smaller, close to 2 cm, but one tumor measured 20 cm in the resected specimen. Most tumors were moderately differentiated HCC with presence of microvascular or macrovascular invasion in 7 patients (29%). Twenty-three patients underwent R0 resection. One patient had a positive margin while undergoing repeat resection of a known recurrence. Fourteen patients underwent major hepatectomy, defined as greater than three Couinard segments [25], and 10 patients underwent minor hepatectomy or wedge resections less than three Couinard segments. None of the patients had previously received either neoadjuvant chemotherapy or neoadjuvant radiation. There were three patients that were positive for Hepatitis C Virus; two of these patients' histologies were classified as steatosis and one patient was designated as having a background of cirrhosis. Two patients died of post-operative complications, one from a myocardial infarction and one from aspiration pneumonia. Three patients were lost to follow up. For the remaining 19 patients, the adjuvant therapies consisted of a heterogeneous mix of chemotherapy, hepatic arterial therapy with either doxorubicin or yttrium-90 microspheres, radiofrequency ablation, repeated surgical resection or no therapy at all. Ten patients are currently without evidence of disease to date. One patient is alive with peritoneal recurrence after nearly 40 months of disease free survival and is now on sorafenib as an adjuvant treatment. Eight patients died of their disease; four of which recurred in the liver only and four had both hepatic recurrences and extrahepatic distant disease in the lungs. Of the patients with known follow up and advanced background histology of either steatohepatitis or cirrhosis (*n* = 15), 8 (53.3%) had a recurrence and 7 (46.7%) went on to die of their disease. The histopathologic grouping and clinical background were summarized in Table 1. The representative histological changes of PT, ST, NASH, CR, and HCC by H&E and Picro-Sirius Red Staining were shown in Figure 1.

**Table 1:** Histologic groupings

	PT	ST	NASH	CR	HCC
Subjects	3	4	9	8	24
(male: female)	1:2	4:0	3:6	5:3	13:11
Median age (yr)	59	59	67	67	67
BMI (kg/m^2^)	27.3	25.4	28.4	26.2	27.37
Median Tumor Size (cm)	8.67 [2, 20]	5.75 [2, 8]	8.67 [4, 17]	8.86 [3, 19]	7.75 [2, 20]
Median # Tumors	1	1	1	1	1

The table summarizes data derived from 2 samples from each of 24 patients, with one sample from each patient derived from the liver tumor, the other from adjacent liver tissue. PT: peritumoral tissue adjacent HCC; ST: steatosis; NASH: steatohepatitis; CR: cirrhosis; HCC: hepatocellular carcinoma.

### Detection of increased FGF19 signaling in HCC

**Figure 2:**
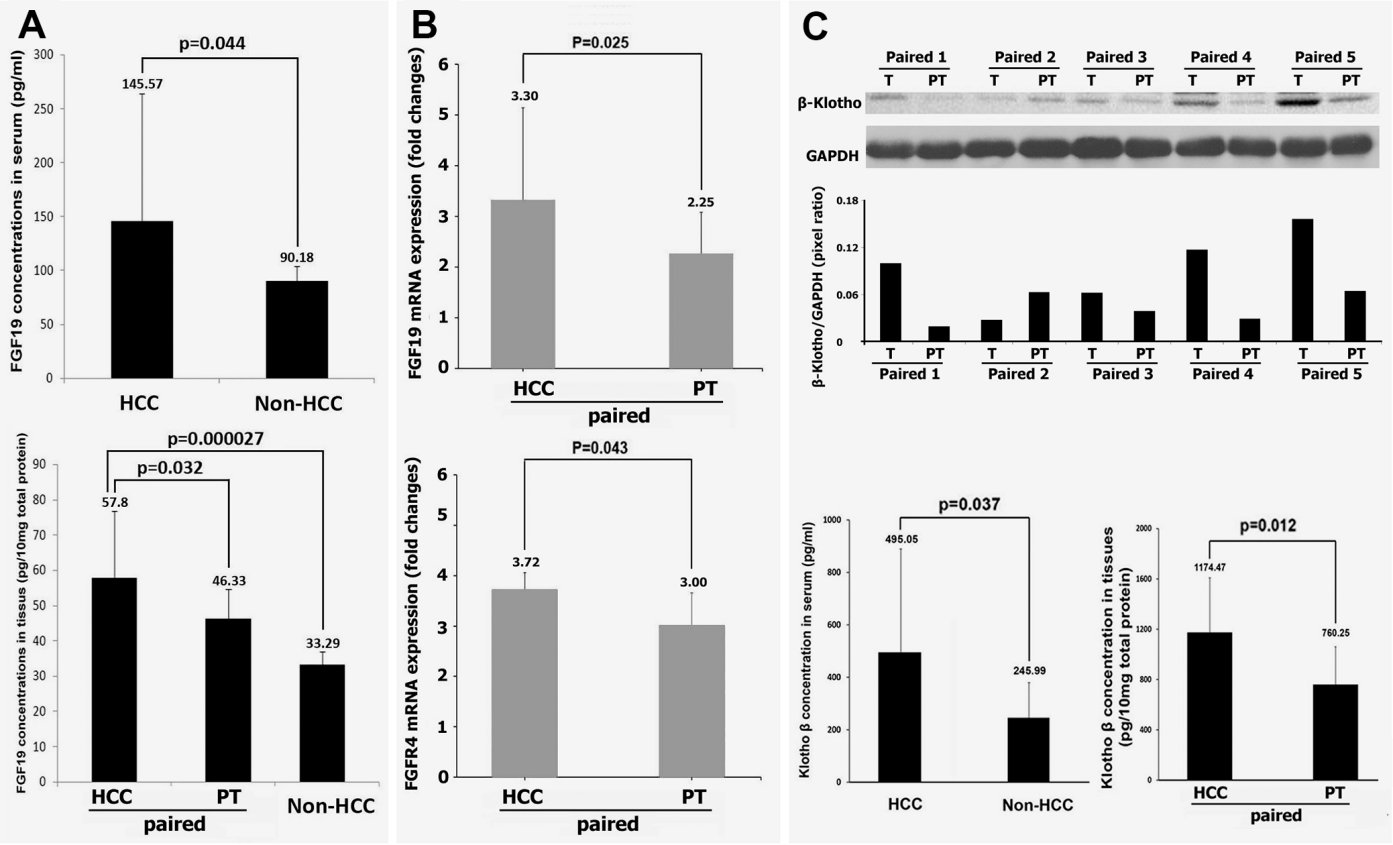
Expressions of FGF19, FGFR4 and of β-Klotho in HCC (**A**) the protein levels of FGF19 in serum from 24 HCC patients and 6 non-HCC controls, and in the tissues from 24 paired HCC-peritumoral and 6 non-HCC controls by ELISA analysis. (**B**) the mRNA levels of FGF19 and FGFR4 from 24 paired HCC-peritumoral tissues. (**C**) upper: Representative Western blot for β-Klotho protein detection of 5 paired tissues from HCC patients. Lower: the protein levels of β-Klotho in serum from 24 HCC patients and 6 non-HCC controls, and in the tissues from 24 paired HCC-peritumoral tissues by ELISA analysis. T: HCC tumor tissue; PT: peritumoral tissues.

As we know, FGF19 along with FGF21 and FGF23 are the three “endocrine FGFs” that can be released into the bloodstream to act throughout the body [13]. We firstly determined the FGF19 protein levels in serum from HCC and non-HCC. The results indicated that FGF19 level was significantly increased in the HCC patients' serum (145.57 pg/ml ± 118.72) compared to non-HCC controls (90.18 pg/ml ± 13.88, *p* = 0.044 vs HCC). Because FGF19 is produced primarily in the ileum and signals in hepatocytes to regulate liver bile acid metabolism [13], we further analyzed the FGF19 level in liver tissue. Levels of FGF19 were determined not only in HCC and non-HCC tissues, but also in the paired peritumoral tissues. Interestingly, these results indicated that FGF19 level was significantly increased in the HCC tissues (57.80 pg/10 mg total protein ± 4.39) compared to both non-HCC tissues (33.29 pg/10 mg total protein ± 1.53, *p* = 0.000027 vs. HCC) and paired peritumoral tissues (46.33 pg/10 mg total protein ± 2.53, *p* = 0.032 vs. HCC), by ELISA analysis (Figure 2A). Although FGF19 was produced primarily in the ileum, it could be autocrined by hepatocellular under cholestatic conditions and peritumoral tissues cirrhosis [17]. To test if FGF19 could be produced in liver, the mRNA level of FGF19 as well as its receptor FGFR4 was determined in HCC and paired peritumoral tissues. The results indicated that FGF19 mRNA expression was significantly increased in the HCC tissues (3.30 ± 1.82) compared to paired peritumoral tissues (2.25 ± 0.82, *p* = 0.025 vs. HCC). FGFR4 mRNA expression was also significantly increased in the HCC tissues (3.72 ± 2.34) compared to paired peritumoral tissues (3.00 ± 0.64, *p* = 0.043 vs. HCC) (Figure 2B). Because a functional FGF19/FGFR4 signaling must require the co-receptor β-Klotho to work together with the receptor FGFR4, therefore β-Klotho protein levels were further studied in the 24 paired tissues as well as serum. Compared to their paired peritumoral tissues, increased β-Klotho protein levels in HCC tissues were found in 19 patients by Western blot analysis, while 2 patients showed decreased and 3 patients showed unchanged in their HCC tissues. The representative data of western blot analysis were shown in Figure 2C. The increased β-Klotho protein levels in HCC tissues and serum were further confirmed by ELISA analysis. As shown in Figure 2C, increased β-Klotho protein levels in HCC tissues were consistent to the Western blot results. The serum β-Klotho protein level was also significantly increased in the HCC patients (495.05 pg/ml ± 394.22) compared to non-HCC patients (245.99 pg/ml ± 134.46, *p* = 0.037 vs. HCC) by ELISA analysis (Figure 2C).

### In situ identification of protein expressions of FGF19, FGFR4, and EpCAM

**Figure 3:**
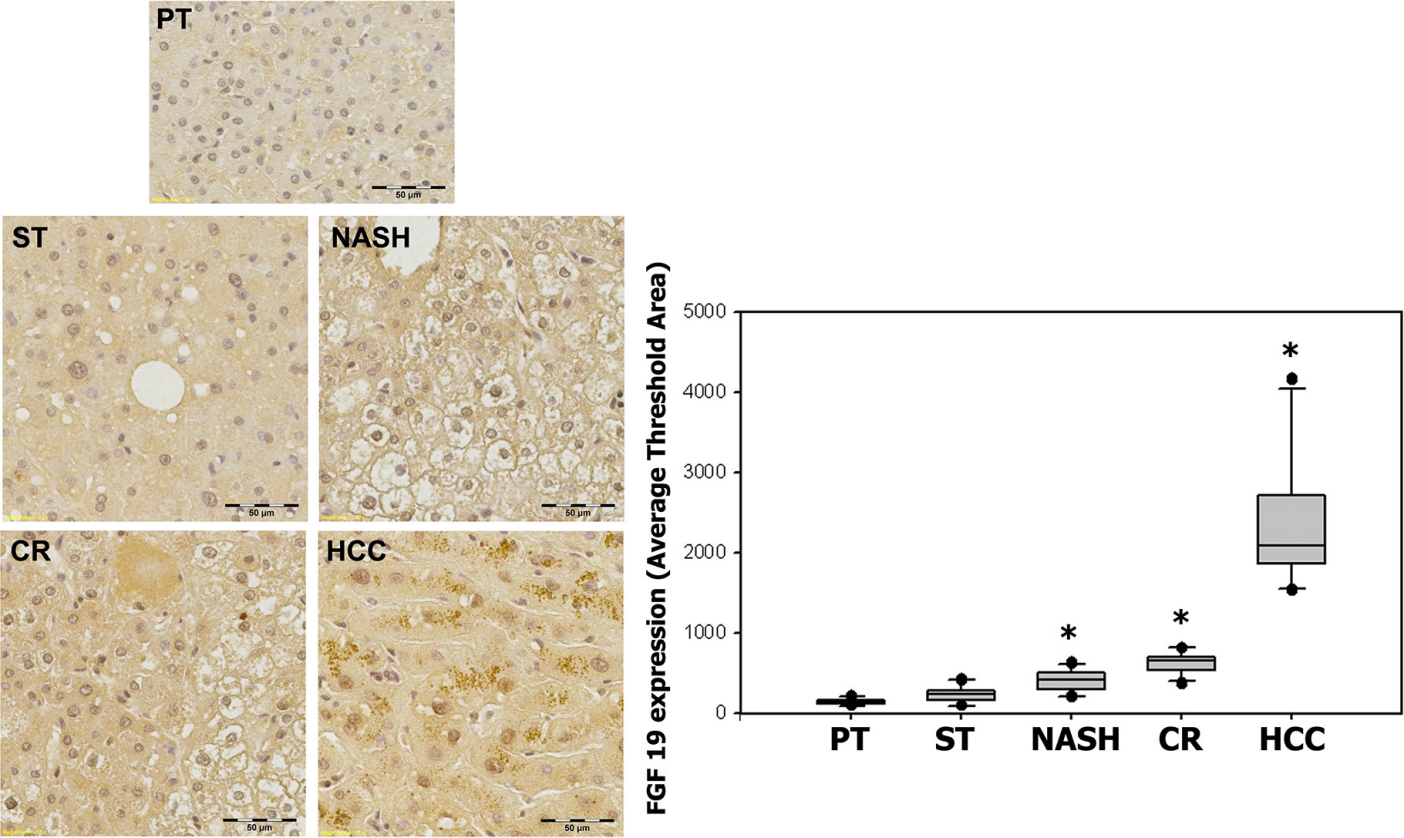
Expression of FGF19 in ST-NAHS-cirrhosis-HCC progression Representative immunohistochemical staining for FGF19 and the computer quantification of FGF19 expression from different stages. Magnification: 200×, the positive staining shown as brown color. PT: peritumoral tissues; ST: steatosis with diffuse lipid deposition; NASH: lipid deposition with inflammatory cells infiltration; CR: cirrhosis with regenerative nodule; HCC: hepatocellular carcinoma. *p < 0.05 vs PT.

The results from the analysis of serum and tissues encouraged us to further analyze the histological distributions of FGF19 and its receptor FGFR4 as well as EpCAM, a potential cancer stem cell marker, corresponding to the clinical histological stages, including ST, NASH, CR and HCC. The result showed that FGF19 expression appeared to increase significantly with the histological severity of liver disease, showing a trend of FGF19 expression in specimens with ST (224.13 ± 115.68, *p* = 0.087), NASH (413.99 ± 159.55, *p* = 0.002), CR (613.35 ± 157.29, *p* < 0.0001) and HCC (2507.28 ± 831.10, *p* = 0.0001) as compared with the paired peritumoral tissues (142.96 ± 41.32). Statistical analysis indicated a significant positive correlation between FGF19 expression and histopathologic changes from ST to HCC (*r* = 0.968) (Figure 3).

**Figure 4:**
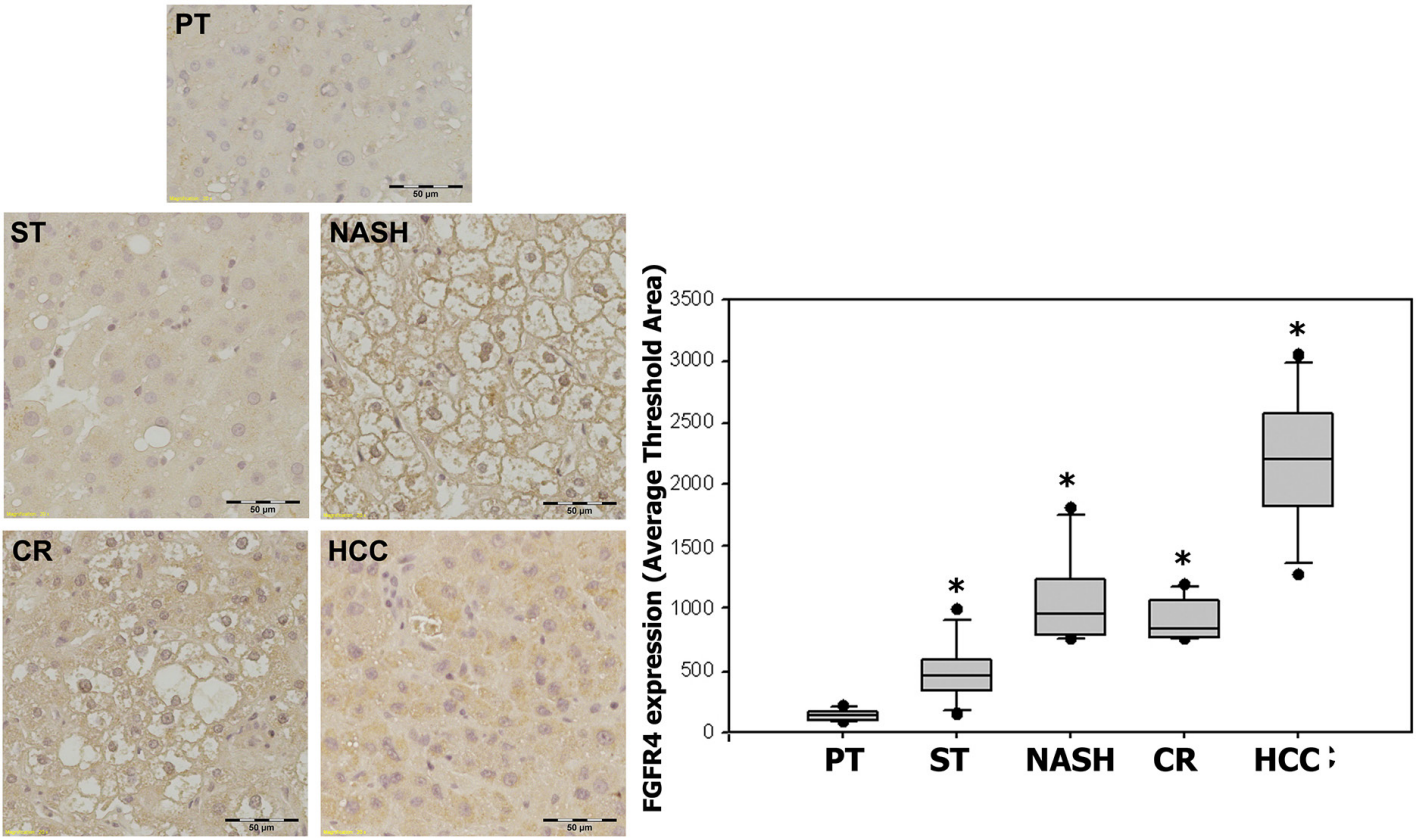
Expression of FGFR4 in ST-NAHS-cirrhosis-HCC progression Representative immunohistochemical staining for FGFR4 and the computer quantification of FGFR4 expression from different stages. Magnification: 200×, the positive staining shown as brown color. PT: peritumoral tissues; ST: steatosis with diffuse lipid deposition; NASH: lipid deposition with inflammatory cells infiltration; CR: cirrhosis with regenerative nodule; HCC: hepatocellular carcinoma. *p < 0.05 vs PT.

The expression of FGF19's corresponding receptor FGFR4 showed statistically significant increases in specimens with ST (539.88 ± 258.37, *p* = 0.004), NASH (1142.52 ± 397.49, *p* = 0.0004), CR (974.96 ± 170.25, *p* < 0.0001) and HCC (2099.32 ± 594.80, *p* < 0.0001) as compared with the paired peritumoral tissues (156.27 ± 47.57). Likewise, FGFR4 expression appeared to increase with the histological severity of hepatic pathology and there was also a significant positive correlation between FGFR4 expression and the histopathologic changes from ST to HCC (*r* = 0.93) (Figure 4).

**Figure 5:**
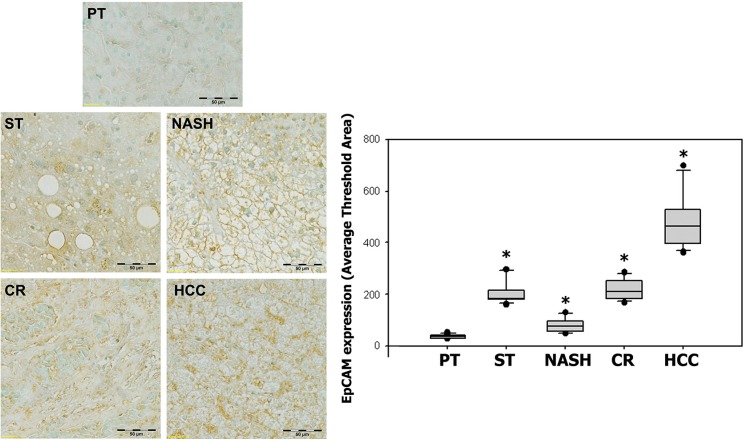
Expression of EpCAM in ST-NAHS-cirrhosis-HCC progression Representative immunohistochemical staining for EpCAM and the computer quantification of EpCAM expression from different stages. Magnification: 200×, the positive staining shown as brown color. PT: peritumoral tissues; ST: steatosis with diffuse lipid deposition; NASH: lipid deposition with inflammatory cells infiltration; CR: cirrhosis with regenerative nodule; HCC: hepatocellular carcinoma.*p < 0.05 vs PT.

EpCAM expression also showed significant increases in specimens with ST (214.45 ± 50.06, *p* < 0.0001), NASH (77.40 ± 28.49, *p* = 0.007), CR (213.74 ± 25.22, *p* < 0.0001) and HCC (483.75 ± 119.92, *p* < 0.0001) as compared with paired peritumoral tissues (38.44 ± 7.31). As previously seen with both FGF19 and FGFR4, EpCAM expression also appeared to increase with the histological severity of inflammation and liver disease, demonstrating a significant positive correlation between EpCAM expression and histopathologic changes from ST to HCC (*r* = 0.947) (Figure 5).

**Figure 6:**
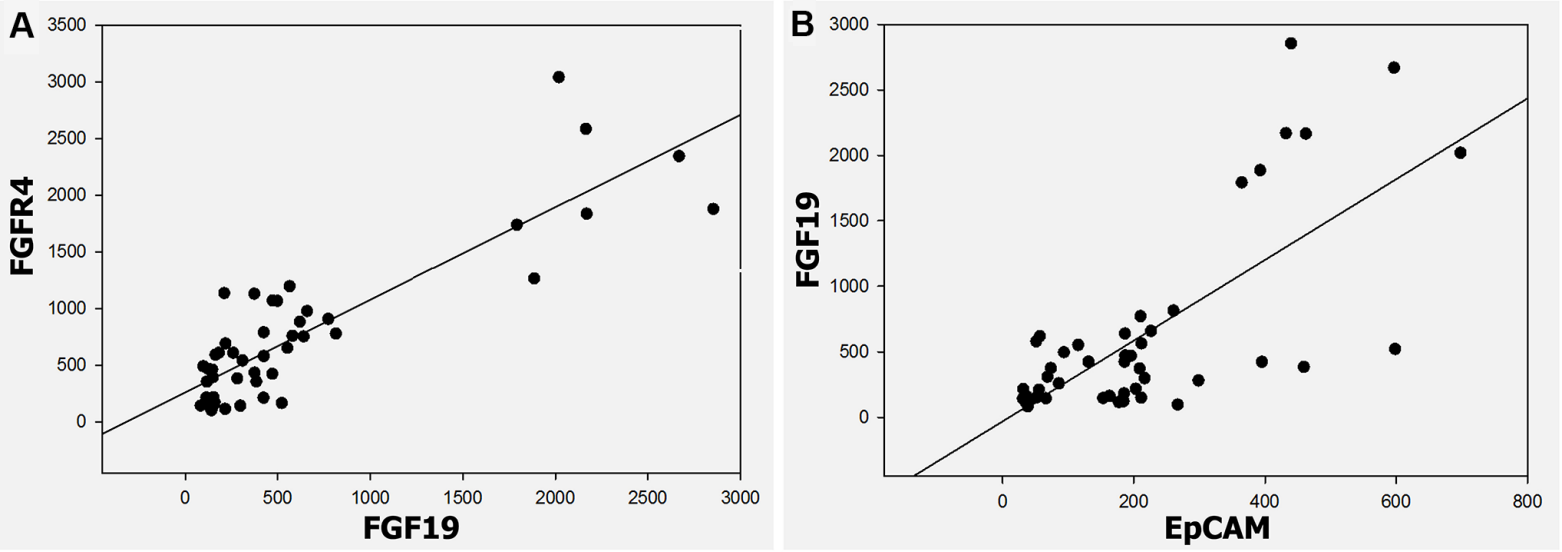
Correlation between FGF19 and FGFR4, FGF19 and EpCAM in ST-NAHS-cirrhosis-HCC progression (**A**) Positive correlation of FGF19 and FGFR4 expression in HCC (*r* = 0.79) *(p* < 0.001). (**B**) Positive correlation of FGF19 and EpCAM expression in HCC (*r* = 0.852) (*p* < 0.001).

The data from FGF19 expression with FGFR4 and EpCAM expression from the 24 immunohistochemically stained HCC sections were used to determine the correlation between FGF19 and its receptor FGFR4 and between the cancer stem cell marker EpCAM, respectively, in the ST-NASH-CR- HCC sequence. A significant positive correlation between FGF19 expression and FGFR4 expression was determined in the HCC tissues (*r* = 0.79; *p* < 0.001) (Figure 6A). Additionally, a significant positive correlation between FGF-19 expression and EpCAM expression was determined in the HCC tissues (*r* = 0.852; *p* < 0.001) (Figure 6B).

## DISCUSSION

The results from this study demonstrate a significant increase in FGF19 in serum and hepatic tissues from HCC patients. The overexpression of FGF19, FGFR4, and EpCAM is seen within a fatty liver microenvironment progressing to HCC when compared to the paired peritumoral liver tissue immediately adjacent to the resected HCC. This increase in FGF19/FGR4 expression in liver tissues was seen overall as a progression of steatosis to HCC.

These findings are consistent with recent reports that FGF19 and FGFR4 may function within the liver to promote growth and proliferation in HCC [18, 26]. Our study revealed that as the worse histopathologic changes were in the liver, there was a corresponding rise in the levels of FGF19 and FGFR4. Mounting evidence from both experimental and epidemiological studies indicates that several pathophysiological mechanisms link fatty liver and hepato-carcinogenesis [7, 27]. The combination of systemic insulin resistance, local chronic inflammation, and oxidative stress cultivate an environment of dysplasia, namely cirrhosis. In cirrhotic liver, changes in fat metabolism associated with the activation of adipocyte-like pathways are thought to be involved in neoplastic transformation [28]. Cirrhosis is present in the vast majority of HCC patients and constitutes the largest single risk factor for development of hepatocellular carcinoma. We suspect that the FGF19 and FGFR4 were up-regulated in response to the underlying inflammatory changes. This chronic state of inflammation predisposing to malignant transformation has previously been reported as a response to insulin resistance within the fatty liver microenvironment [29]. Therefore fatty changes can be regarded as dysplasia or a pre-neoplastic lesion of HCC. Inflammatory changes due to underlying NASH, an aggressive histologic variant of nonalcoholic fatty liver disease (NAFLD), will likely lead to increased numbers of patients with HCC in the near future due to the prevalence of obesity and NAFLD. It has even been speculated that NASH will overtake HCV/HBV as the most common associated risk factor for HCC in the next decade due to the prevalence of obesity and NAFLD [30].

The knowledge of hepatic oncogenic activation and signaling pathways for proliferation, de-differentiation, invasion, and metastasis has led to the discovery of hepatic cancer stem cells (CSC) in HCC. In our study, all of the human liver tissue samples from the surgical resections, including the normal adjacent liver specimens, expressed the FGF19, FGFR4 and EpCAM. But more importantly, the pattern of increasing expression of FGF19, FGFR4 and EpCAM within the HCC, suggests a mechanistic interaction between the fibroblast growth factor/receptor and hepatic cancer stem cells, which has not been described prior to this study. While numerous pathways from altered fat metabolism to inflammation have been described in relation to HCC [31], there seems to be a missing link in understanding the promotion of hepatic progenitor cells within the fatty liver microenvironment. It is known that EpCAM, a cell surface antigen and mediator of cellular adhesion and signaling, is a specific biologic marker for hepatic cancer stem cells [32]. Our data confirmed a lower level of EpCAM expression in peritumoral liver tissue but a high level of expressed in the HCC specimens. Overexpression of EpCAM has been associated with more malignant biological phenotypes as a reflection of the evasive pluripotent cancer stem cell population and worse survival [33, 34]. Our data support the role of FGF19/FGFR4 as a promoter of hepatic stem cells in HCC as seen by the strong correlation between FGF19 and EpCAM. Malignant transformation to HCC within the fatty liver tumor microenvironment is clearly an exceedingly complex process. This study helps to elucidate a relationship between FGF19 and its respective receptor FGFR4 in the promotion of hepatic stem cells as indicated by increased EpCAM within the carcinogenesis sequence of fatty liver to hepatocellular carcinoma in the specimens. Therefore we propose that a simultaneous increase of FGF19/FGFR4 associated with an increased level of EpCAM may help to identify which patients may have a more aggressive and resistant tumor biology requiring multi-drug therapy.

Unfortunately, most HCC patients present with advanced disease and have high rates of recurrence, therefore, early detection and multimodal therapy is of critical importance to improve overall survival. There is a current focus on understanding tumor biology through molecular profiling by either invasive tissue sampling with percutaneous biopsy or surgical resection, or by serologic testing for circulating tumor cells to assess propensity for hematogenous metastasis or recurrence. Previous reports have identified several serum biomarkers for HCC which can be used for diagnosis and prognostic indicators in HCC [35, 36]. Our serum FGF19 data consist to expressions of FGF19/FGFR4 and EpCAM in tissue distributions, and provide further evidence for FGF19 as a marker to evaluate the risk of HCC in patients with fatty liver disease.

It has been demonstrated that efficient FGF19/FGFR4 signaling relies on the expression of β-klotho trans-membrane co-receptor, abundantly expressed in hepatocytes. However, the β-klotho expression in HCC remains unknown. This is the first study that reports β-klotho expression proteins are not only increased in HCC tissue, but also in the serum of HCC patients. The increases of β-klotho protein expression in HCC tissues may be caused by the endoplasmic reticulum (ER) stress in the hepatic tissues which is supported by the studies from previous studies from this institution and others [37, 38]. The increased β-klotho protein expression in HCC serum needs to be further studied to clarify the exact mechanism. In addition, the HCC harboring FGF19 amplification is believed a switch from intestine-driven endocrine to autocrine by hepatocellular signaling in liver under cirrhosis [17]. However, ectopic expression of FGF19 has also been reported during the development of HCC. An ileum-derived FGF15 has been found to contribute to hepatocarcinogenesis [39]. Therefore, the high levels of serum FGF19 in HCC patients could be both ileum-derived and hepatocellular derived by autocrine in the presence of pro-tumorigenic and tumorigenic conditions, including NASH, cirrhosis and HCC, but further study, again, is needed.

The limitation of this study was the small sample size of 24 individual patients, however we were able to identify significant differences in expression when we used the peritumoral liver tissue paired to the HCC tissue for data analysis by ELISA, Western blots and RT-PCR in the study cohort. Nevertheless, this limited preliminary data is compelling and suggests that additional work needs to be done to firmly establish FGF19/FGFR4/EpCAM signaling as a biomarker for HCC risk assessment. In conclusion, FGF19/FGFR4 and EpCAM protein expression is significantly increased in the progression of fatty liver to hepatocellular carcinogenesis. Overexpression of FGF19/FGFR4 significantly correlated with EpCAM as a marker of hepatic cancer stem cells within the fatty-steatosis-cirrhosis-HCC sequence. Further study is needed to clarify this mechanism of action of FGF19/FGFR4 signaling on cancer stem cells within the fatty liver microenvironment as a risk factor for HCC.

## MATERIALS AND METHODS

### Case selection and review

This study was approved by the Institutional Review Board for Human Study at the University of Louisville. The HCC samples were prospectively collected from 24 patients who had undergone liver resection for hepatocellular carcinoma between 2002 and 2013. Tumor tissues along with corresponding peritumoral tissues adjacent to HCC and sera from Hepatocellular Carcinoma (HCC) patients, as well as tissues and sera from non-HCC controls (liver adenoma and hemangioma) were acquired from the James Graham Brown Cancer Center Bio-Repository at University of Louisville following an approved IRB protocol. All of the tissue samples from HCC patients were selected with established diagnoses of peritumoral tissues, steatosis, NASH, cirrhosis, and HCC. The classification of peritumoral tissues (PT) was assigned if there were no abnormalities such as cancerous cells, steatosis, fibrosis, necrosis, inflammatory infiltration, nor lipid drops found in the tissue. The specimen was designated as steatosis (ST) if there was an increase in lipid drops within a background of normal hepatic parenchyma. The specimen was designated as NASH was the assigned histology if there was an infiltration or considerable lipid deposition with additional inflammatory changes. Cirrhosis (CR) was the designated histology if there was loss of portal structures due to fibrotic or significant inflammatory alteration of the tissue. HCC was the designated histology if there were cancerous cells. A microscope examination of the background cellular composition of hepatic tissue confirmed the diagnoses of PT, ST, NASH, CR and HCC on these liver tissues reviewed by two pathologists independently, blinded to the subject's clinical history. The tissue specimens were graded on their histologic structure of the portal tracts, central veins, and hepatic parenchyma. No preoperative chemo- or radiotherapy had been administered to the patients, nor had any patient received a non-steroidal anti-inflammatory regimen. For the study of protein levels FGF19 and β-Klotho in serum and tissue, the samples were from 24 HCC patients as well as 6 non-HCC controls (liver adenoma and hemangioma). For the immunohistochemical study, the tissue samples were from the same 24 HCC patients, the HCC tissue samples consisting of peritumoral hepatic tissues adjacent to the HCC in the resected hepatectomy specimens was examined and compared to their malignant counterpart.

### Human FGF19 and β-Klotho ELISA assays

The levels of serum FGF19 and β-Klotho and tissue FGF19 and β-Klotho were determined using Human FGF19 ELISA assay kits (R&D Systems, Inc. Minneapolis, MN) according to the manufacturer's instructions. For the FGF19 measurement, in brief, the homogenized tissues and serum were collected using EDTA as an anticoagulant and were centrifuged for 15 minutes at 1000 × g within 30 minutes of collection. Both tissue and serum sample were standardized corresponding to the total protein concentration. The FGF-19 standards were reconstituted with 1.0 mL of deionized water. Assay Diluent RD1S was added into each well of the provided 96-well plate. Then, standard and sample were added and incubated for 2 hours at room temperature. After washing, 200 μL of FGF-19 conjugate was added and incubated for 2 hours at room temperature. Substrate Solution was added, incubated for 30 minutes at room temperature, and stopped using Stop Solution. Optical density was determined using a microplate reader at 450 nm. The FGF19 concentrations were calculated according to the standard curve. Similarly, the β-Klotho concentrations in serum and tissues were measured using β-Klotho kit.

### Tissue array and histology

The tissue array procedures were performed concurrently to allow simultaneous analysis and experimental uniformity. With this technology, the tissue samples were treated in an identical manner for histochemical and immunohistochemical stains to avoid substantial slide-to-slide variability associated with processing the individual slides. This tissue array allows the cohort to be analyzed in one batch on a single slide, thus, variables such as antigen retrieval, temperature, incubation times, washing procedure, and reagent concentration were standardized for the entire cohort. In this study, a total of 48 tissue samples were examined for measurement of FGF19, FGFR4, and EpCAM. The 24 control samples consisted of the same 24 patients' normal adjacent tissue that was within the resected liver specimen. The samples of HCC tissues as well as paired peritumoral liver tissues were fixed in 10% buffered formalin for 48 hours and processed for paraffin embedding. The number of spots on a single block was designed as three pairs for each tissue array, and each piece of analyzed tissue was 5–8 mm in size to allow for a larger review area. After embedding in paraffin, 5-μm sections were mounted onto glass slides and stained with hematoxylin and eosin (H&E) for histopathologic analysis. The Picro-Sirius Red Stain Kit specific for connective tissue was used for the histological visualization of collagen I and III fibers in the tissue sections.

### Immunohistochemical assay for FGF19, FGFR4 and EpCAM

The *in situ* FGF19, FGFR4 and EpCAM protein expressions were determined in the tissue by using an immunohistochemical assay. Staining was carried out on the paraffin-embedded tissues using the DAKO EnVision^TM^+System Kit (DAKO Corporation, Carpinteria, CA) according to the manufacturer's instructions. In brief, the sections were deparaffinized and hydrated, then the slides were washed with TRIS-buffer. Peroxidase blocking was performed for 5 minutes. After rewashing, the slides were incubated separately with the monoclonal mouse FGF19 antibody (1:100), FGFR4 antibody (1:100), or EpCAM antibody (1:100) (SantaCruz Biotechnology Inc, CA) for 30 minutes at room temperature. The slides were rinsed, and the specimens were incubated with labeled polymer for 30 minutes at room temperature. Then the chromogenic substrate diaminobenzidine was added as a visualization reagent. Finally, the slides were counterstained with hematoxylin for FGF19 and FGFR4, with methyl green for EpCAM. A negative control was included in each run.

### Computer image analysis

A computer image analysis was performed to quantify the expressions of FGF19, FGFR4 and EpCAM in the 24 samples diagnosed with ST, NASH, CR, HCC, and in the paired samples of peritumoral liver tissues. The imaging fields were chosen randomly from various section levels to ensure objectivity of sampling. Five imaging fields were scanned for each specimen sample. All digital images were acquired with the Olympus 1 × 51 microscope at 40× magnification using the Olympus DP72 digital camera via the cellSens Dimention imaging system (Olympus, Pittsburgh, PA) and stored as JPG data files, with fixed resolutions of 200 pixels/inch. The acquired color images from the immunohistochemical staining were defined at a standard threshold according to the software specification. The computer program then quantified the threshold area represented by color images. FGF19, FGFR4 and EpCAM protein expressions were defined by the averages of threshold area in acquired color images.

### RT-PCR assay for FGF19 and FGFR4

Total RNA was extracted using the TRIzol reagent (Invitrogen, CA). First-strand complimentary DNA (cDNA) was synthesized from total RNA according to the manufacturer's protocol for the RNA PCR kit (Promega, Madison, WI). Quantitative PCR was carried out using the ABI 7300 real-time PCR system (Applied Biosystems, Carlsbad, CA). Template cDNAs in each testing sample and the reference were tested in triplicate for quantitative expression levels of FGF19 and FGFR4 genes and housekeeping gene GAPDH on 96 well plates designed for ABI PRISM^®^ 3700 series thermocyclers. The delta-delta crossing threshold (ddCt) method was used to determine fold changes in the testing samples compared to the reference of averaged GAPDH for normalization.

### Western blot for β-Klotho

Protein levels for the biomarkers were semi-quantified by Western blot analysis as described previously. Electrophoresis was performed on 12% SDS-PAGE gel and the proteins were transferred to nitrocellulose membranes. Membranes were incubated with primary antibody of β-Klotho (Abcam, Inc, Cambridge, MA) overnight at 4°C and with secondary antibody for 1 hr at room temperature. The antigen-antibody complexes were then visualized using ECL (Amersham, Piscataway, NJ).

### Statistical analysis

The results are expressed as mean values ± standard deviation (SD). Analysis of variance was performed using SigmaPlot 11.0 (Systat Software Inc, San Jose, CA) The data were analyzed by analysis of variance (ANOVA) and Newman-Keuls' Multiple-Comparison Test were used to determine the differences in FGF19, FGFR4 and EpCAM expressions between the pooled samples from each histologic stage of carcinogenesis beginning with fatty liver progressing to steatohepatitis, cirrhosis, and finally hepatocellular carcinoma. Spearman rank correlation coefficient (R) was used to analyze the correlation between FGF19 and FGFR4, and correlation between FGF19 and EpCAM, with the various histologic stages. *p* values less than 0.05 were considered statistically significant.

## References

[1] El-Serag HB (2011). Hepatocellular carcinoma. N Engl J Med.

[2] Jemal A, Bray F, Center MM, Ferlay J, Ward E, Forman D (2011). Global cancer statistics. CA Cancer J Clin.

[3] Li T, Qin LX, Gong X, Zhou J, Sun HC, Wang L, Qiu SJ, Ye QH, Fan J (2014). Clinical characteristics, outcome, and risk factors for early and late intrahepatic recurrence of female patients after curative resection of hepatocellular carcinoma. Surgery.

[4] Onnerhag K, Nilsson PM, Lindgren S (2014). Increased risk of cirrhosis and hepatocellular cancer during long-term follow-up of patients with biopsy-proven NAFLD. Scand J Gastroenterol.

[5] Schutte K, Schulz C, Poranzke J, Antweiler K, Bornschein J, Bretschneider T, Arend J, Ricke J, Malfertheiner P (2014). Characterization and prognosis of patients with hepatocellular carcinoma (HCC) in the non-cirrhotic liver. BMC gastroenterol.

[6] Scalera A, Tarantino G (2014). Could metabolic syndrome lead to hepatocarcinoma non-alcoholic fatty liver disease?. World J Gastroenterol.

[7] Siegel AB, Zhu AX (2009). Metabolic syndrome and hepatocellular carcinoma: two growing epidemics with a potential link. Cancer.

[8] Regimbeau JM, Colombat M, Mognol P, Durand F, Abdalla E, Degott C, Degos F, Farges O, Belghiti J (2004). Obesity and diabetes as a risk factor for hepatocellular carcinoma. Liver Transpl.

[9] Lazo M, Hernaez R, Eberhardt MS, Bonekamp S, Kamel I, Guallar E, Koteish A, Brancati FL, Clark JM (2013). Prevalence of nonalcoholic fatty liver disease in the United States: the Third National Health and Nutrition Examination Survey, 1988–1994. Am J Epidemiol.

[10] Kim CH, Younossi ZM (2008). Nonalcoholic fatty liver disease: a manifestation of the metabolic syndrome. Cleve Clin J Med.

[11] Tateishi R, Okanoue T, Fujiwara N, Okita K, Kiyosawa K, Omata M, Kumada H, Hayashi N, Koike K (2015). Clinical characteristics, treatment, and prognosis of non-B, non-C hepatocellular carcinoma: a large retrospective multicenter cohort study. J Gastroenterol.

[12] Kikuchi L, Oliveira CP, Carrilho FJ (2014). Nonalcoholic fatty liver disease and hepatocellular carcinoma. Biomed Res Int.

[13] Potthoff MJ, Kliewer SA, Mangelsdorf DJ (2012). Endocrine fibroblast growth factors 15/19 and 21: from feast to famine. Genes Dev.

[14] Kir S, Kliewer SA, Mangelsdorf DJ (2011). Roles of FGF19 in liver metabolism. Cold Spring Harb Symp Quant Biol.

[15] Jones SA (2012). Physiology of FGF15/19. Adv Exp Med Biol.

[16] Nicholes K, Guillet S, Tomlinson E, Hillan K, Wright B, Frantz GD, Pham TA, Dillard-Telm L, Tsai SP, Stephan JP, Stinson J, Stewart T, French DM (2002). A mouse model of hepatocellular carcinoma: ectopic expression of fibroblast growth factor 19 in skeletal muscle of transgenic mice. Am J Pathol.

[17] Lin BC, Desnoyers LR (2012). FGF19 and cancer. Adv Exp Med Biol.

[18] Miura S, Mitsuhashi N, Shimizu H, Kimura F, Yoshidome H, Otsuka M, Kato A, Shida T, Okamura D, Miyazaki M (2012). Fibroblast growth factor 19 expression correlates with tumor progression and poorer prognosis of hepatocellular carcinoma. BMC cancer.

[19] Hyeon J, Ahn S, Lee JJ, Song DH, Park CK (2013). Expression of fibroblast growth factor 19 is associated with recurrence and poor prognosis of hepatocellular carcinoma. Dig Dis Sci.

[20] Wu X, Ge H, Lemon B, Vonderfecht S, Weiszmann J, Hecht R, Gupte J, Hager T, Wang Z, Lindberg R, Li Y (2010). FGF19-induced hepatocyte proliferation is mediated through FGFR4 activation. J Biol Chem.

[21] Wu X, Li Y (2012). Understanding the structure-function relationship between FGF19 and its mitogenic and metabolic activities. Adv Exp Med Biol.

[22] Yamashita T, Ji J, Budhu A, Forgues M, Yang W, Wang HY, Jia H, Ye Q, Qin LX, Wauthier E, Reid LM, Minato H, Honda M (2009). EpCAM-positive hepatocellular carcinoma cells are tumor-initiating cells with stem/progenitor cell features. Gastroenterology.

[23] Tomuleasa C, Soritau O, Rus-Ciuca D, Pop T, Todea D, Mosteanu O, Pintea B, Foris V, Susman S, Kacso G, Irimie A (2010). Isolation and characterization of hepatic cancer cells with stem-like properties from hepatocellular carcinoma. J Gastrointestin Liver Dis.

[24] Muramatsu S, Tanaka S, Mogushi K, Adikrisna R, Aihara A, Ban D, Ochiai T, Irie T, Kudo A, Nakamura N, Nakayama K, Tanaka H, Yamaoka S (2013). Visualization of stem cell features in human hepatocellular carcinoma reveals *in vivo* significance of tumor-host interaction and clinical course. Hepatology.

[25] Couinaud C (1980). Definition of hepatic anatomical regions and their value during hepatectomy (author's transl). Chirurgie.

[26] Ho HK, Pok S, Streit S, Ruhe JE, Hart S, Lim KS, Loo HL, Aung MO, Lim SG, Ullrich A (2009). Fibroblast growth factor receptor 4 regulates proliferation, anti-apoptosis and alpha-fetoprotein secretion during hepatocellular carcinoma progression and represents a potential target for therapeutic intervention. J Hepatol.

[27] Starley BQ, Calcagno CJ, Harrison SA (2010). Nonalcoholic fatty liver disease and hepatocellular carcinoma: a weighty connection. Hepatology.

[28] Terasaki S, Kaneko S, Kobayashi K, Nonomura A, Nakanuma Y (1998). Histological features predicting malignant transformation of nonmalignant hepatocellular nodules: a prospective study. Gastroenterology.

[29] Schreuder TC, Marsman HA, Lenicek M, van Werven JR, Nederveen AJ, Jansen PL, Schaap FG (2010). The hepatic response to FGF19 is impaired in patients with nonalcoholic fatty liver disease and insulin resistance. Am J Physiol Gastrointest Liver Physiol.

[30] Wong RJ, Cheung R, Ahmed A (2014). Nonalcoholic steatohepatitis is the most rapidly growing indication for liver transplantation in patients with hepatocellular carcinoma in the U. S. Hepatology.

[31] Alzahrani B, Iseli TJ, Hebbard LW (2014). Non-viral causes of liver cancer: does obesity led inflammation play a role?. Cancer lett.

[32] Oishi N, Yamashita T, Kaneko S (2014). Molecular biology of liver cancer stem cells. Liver cancer.

[33] Guo Z, Li LQ, Jiang JH, Ou C, Zeng LX, Xiang BD (2014). Cancer stem cell markers correlate with early recurrence and survival in hepatocellular carcinoma. World J Gastroenterol.

[34] Chan AW, Tong JH, Chan SL, Lai PB, To KF (2014). Expression of stemness markers (CD133 and EpCAM) in prognostication of hepatocellular carcinoma. Histopathology.

[35] Witjes CD, van Aalten SM, Steyerberg EW, Borsboom GJ, de Man RA, Verhoef C, Ijzermans JN (2013). Recently introduced biomarkers for screening of hepatocellular carcinoma: a systematic review and meta–analysis. Hepatol Int.

[36] Wong KF, Xu Z, Chen J, Lee NP, Luk JM (2013). Circulating markers for prognosis of hepatocellular carcinoma. Expert Opin Med Diagn.

[37] Zhang Q, Li Y, Liang T, Lu X, Zhang C, Liu X, Jiang X, Martin RC, Cheng M, Cai L (2015). ER stress and autophagy dysfunction contribute to fatty liver in diabetic mice. Int J Biol Sci.

[38] Dong K, Li H, Zhang M, Jiang S, Chen S, Zhou J, Dai Z, Fang Q, Jia W (2015). Endoplasmic reticulum stress induces up-regulation of hepatic beta-Klotho expression through ATF4 signaling pathway. Biochem Biophys Res Co.

[39] Uriarte I, Latasa MU, Carotti S, Fernandez-Barrena MG, Garcia-Irigoyen O, Elizalde M, Urtasun R, Vespasiani-Gentilucci U, Morini S, de Mingo A, Mari M, Corrales FJ, Prieto J (2015). Ileal FGF15 contributes to fibrosis-associated hepatocellular carcinoma development. Int J Cancer.

